# An Account of Diseases in an Eastern District of London, from September 20, to October 20, 1808

**Published:** 1808-11

**Authors:** 


### *An Account of Diseases in an Eastern District of London, from September 20, to October 20, 1808.*

| | |
|---|---|
| **Acute Diseases.** | |
| Typhus - - - | 3 |
| Cholera - - - | 7 |
| Ephemera - - | 3 |
| Icterus - - - | 1 |
| Rheumatismus Acutus | 2 |
| **Chronic Diseases.** | |
| Tussis - - - | 4 |
| Tussis cum Dyspnœa | 7 |
| Hydrothorax - - | 1 |
| Anasarca - - | 3 |
| Ascites - - - | 2 |
| Enterodynia - - | 5 |
| Diarrhœa - - - | 15 |
| Hepatitis Chronica - | 2 |
| Prolapsus Ani - - | 1 |
| Dyspepsia - - | 5 |
| Hemiplegia - | 2 |
| Gonorrhœa Simplex - | 3 |
| Leucorrhœa - - | 5 |
| Amenorrhœa - - | 3 |
| Rheumatismus Chronicus | 5 |
| **Puerperal Diseases.** | |
| Mastodynia - - | 5 |
| Rhagus Papillæ - | 2 |
| Menorrhagia Lochialis | 3 |
| Prolapsus Vaginæ - | 1 |
| Diarrhœa - - | 7 |
| **Infantile Diseases.** | |
| Tabes Mesenterica - | 2 |
| Convulsio - - | 1 |
| Ophthalmia - - | 2 |
| Ophthalmia Purulenta | 1 |
| Aphthæ - - - | 4 |

It may be seen by the preceding list, that different complaints of the bowels have formed a considerable proportion of the diseases of the last few weeks; but there are no circumstances respecting them that are particularly worthy of being recorded.

